# “This is not me”; an overview

**DOI:** 10.1192/j.eurpsy.2021.610

**Published:** 2021-08-13

**Authors:** A. Gonzaga Ramírez, C. Capella Meseguer, E. Rodríguez Vázquez, M. Queipo De Llano De La Viuda, G. Guerra Valera

**Affiliations:** 1 Psiquiatría, Hospital Clínico Universitario de Valladolid, Valladolid, Spain; 2 Psiquiatría, HCUV, Valladolid, Spain

**Keywords:** gender dysphoria, Child Psychiatry, gender identity

## Abstract

**Introduction:**

APA describes Gender dysphoria (GD) as the conflict between a person’s physical or assigned gender and the gender with which he/she/they identify. Recently DSM-V renamed gender identity disorder as “gender dysphoria”. This change in terminology removes the ‘pathology’ from being transgender, which is not a mental health condition.

**Objectives:**

To systematically summarise available evidence in this important but less researched field.

**Methods:**

A comprehensive review was carried using the PubMed/ Medline database.

**Results:**

Formal epidemiological studies of gender dysphoria in children and adolescents have not been conducted. The true prevalence os gender dysphoria is unknown around the world because of the varying definitions, different cultural norms and lack of data. Individuals who identify as transgender are vulnerable, and have higher rates of psychiatric comorbility compared with the general population. Gender dysphoria, gender identity disorder or transsexualism is a psychological condition that requires care and multiple health professionals.

**Conclusions:**

The natural history of gender identity for children who express gender nonconforming or transgender identities is an area of active research. In addition, there is a lack of guidelines to approach these patients.

